# Surface Characteristics and Catalytic Activity of Copper Deposited Porous Silicon Powder

**DOI:** 10.3390/ma7127737

**Published:** 2014-12-04

**Authors:** Muhammad Yusri Abdul Halim, Wei Leng Tan, Noor Hana Hanif Abu Bakar, Mohamad Abu Bakar

**Affiliations:** Nanoscience Research Laboratory, School of Chemical Sciences, Universiti Sains Malaysia, Penang 11800, Malaysia; E-Mails: yusri17@yahoo.com (M.Y.A.H.); weileng728@gmail.com (W.L.T.); bmohamad@usm.my (M.A.B.)

**Keywords:** porous silicon powder, Cu particles, surface properties, p-nitrophenol

## Abstract

Porous structured silicon or porous silicon (PS) powder was prepared by chemical etching of silicon powder in an etchant solution of HF: HNO_3_: H_2_O (1:3:5 v/v). An immersion time of 4 min was sufficient for depositing Cu metal from an aqueous solution of CuSO_4_ in the presence of HF. Scanning electron microscopy (SEM) analysis revealed that the Cu particles aggregated upon an increase in metal content from 3.3 wt% to 9.8 wt%. H_2_-temperature programmed reduction (H_2_-TPR) profiles reveal that re-oxidation of the Cu particles occurs after deposition. Furthermore, the profiles denote the existence of various sizes of Cu metal on the PS. The Cu-PS powders show excellent catalytic reduction on the p-nitrophenol regardless of the Cu loadings.

## 1. Introduction

Porous silicon (PS) was discovered more than 50 years ago; however, the interest of the scientific community on PS was only triggered in recent years. PS possesses several interesting characteristics. Among its most significant characteristics are its very large specific area, which can reach up to 900 m^2^·cm^−3^, the possibility to modify its pore size and morphology according to various requirements, as well as its biocompatibility and non-toxicity. As such, PS has been incorporated with biological molecules, because it allows the loading of large quantities of such materials. Furthermore, devices that can be deposited in humans without undesirable reactions can also be developed [1]. In addition, PS is widely used in electronic and optic devices [2]. Hence, this illustrates the technological benefits of PS in specific applications.

Recently, the use of PS in the area of catalysis has been gaining more attention. The nature of the chemical surface, porous architecture, as well as the semiconducting properties of PS make it a potential support material for catalytic applications [3]. For instance, the Si-H*_x_* terminated groups on the surface of PS are capable of reducing metal ions without the need for an additional reducing agent [4]. The porous structure of the PS is able to accommodate the as-formed metal particles and control the particle dispersion. Apart from that, PS, which is a semiconductor, may also influence the electronic state of the metal active sites and affect the catalytic properties as a whole. Pioneering works on metal-PS nanocomposites, tailored for the application of catalysis, have been demonstrated by Llorca and coworkers [5]. These researches demonstrated the effectiveness of PS membranes coated with a thin layer of Co_3_O_4_–ZnO for the production of H_2_ by steam reforming of ethanol. The use of metal-supported PS as catalysts was further scrutinized by Polisski [6]. He reported that the prepared Pt-PS and Pd-PS catalysts are highly active and stable towards hydrogenation reactions and CO oxidation. A recent report by Liu* et al.* [7] also showed that the Ag nanoparticles supported on PS chips exert high catalytic reduction on p-nitrophenol. Meanwhile, Yashtulov* et al.* [8] studied the influence of PS’s porosity and conductivity type on the phase/charge state of Pt in Pt/PS nanocatalyst.

To date, research on metal/PS catalysts is still in its infancy, and only a few studies are found in the literature. To the best of our knowledge, there is scarcely any report on Cu-supported PS powder for catalytic applications, particularly for the reduction of p-nitrophenol. Generally, p-nitrophenol is a well-known toxic pollutant, a consequence of the current wide use of pesticides, insecticides and synthetic dyes. Most research related to the reduction of this aromatic compound has focused on the use of noble metals, such as Pd, Au and Ag. In the case of Cu as a catalyst for the reduction of p-nitrophenol, not many works have been reported. Among them are magnetic catalysts, such as Cu-Fe_3_O_4_ [9] and Cu-Fe_3_O_4_ with graphene [10] composites. According to Feng and coworkers [9], the Cu particles played a significant role as a catalyst for the reduction of p-nitrophenol, while Fe_3_O_4_ was inactive. However, Ru and coworkers [10] demonstrated that Fe_3_O_4_ partly contributed to the reaction, too. Other works with regards to this catalytic reaction involve the synthesis of Cu dendrimers [11], Cu-doped TiO_2_ [12], as well as Cu nanorods and nanospheres [13]. In all of these works, it has been shown that Cu can be an effective catalyst; however, this depends on factors, such as its morphology and the materials employed to stabilize it or synthesized with it. Hence, in this study, we prepared PS powder via the chemical etching technique and used it as a reducing agent and support for the deposition of Cu particles. The use of PS provides a novel environment for Cu, which can affect the catalytic reduction of p-nitrophenol. In view of the importance of surface characteristics in relation to catalytic properties, the samples were characterized by scanning electron microscopy (SEM), H_2_-temperature programmed reduction analysis (H_2_-TPR) and the Brunauer–Emmett–Teller (BET) method.

## 2. Results and Discussion

### 2.1. Synthesis of PS

Porous silicon powder was prepared via chemical etching, although other techniques, such as metal-assisted chemical etching [14,15] and electrochemical etching [16], have been reported to produce PS. The technique employed in this work is particularly attractive, because of its simplicity and the presence of readily available oxidizing and corrosive reagents; namely nitric acid (HNO_3_) and water (H_2_O), as well as hydrogen fluoride (HF).

**Figure 1:**
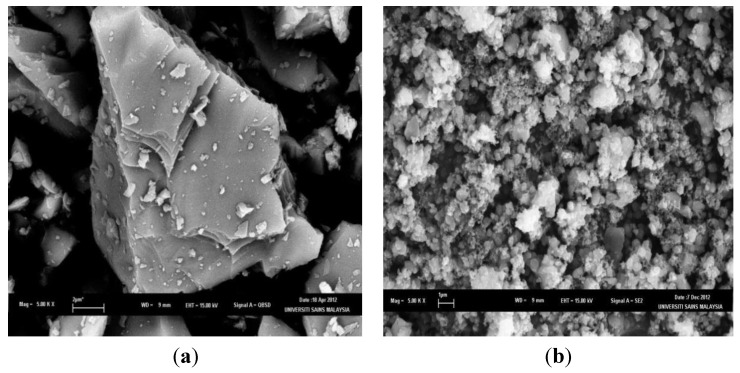
SEM images of (**a**) powdered silicon and (**b**) porous silicon (PS) powder after 4 min of etching.

The morphology of the resulting PS powder was investigated and compared to powdered silicon. A typical image of the powdered silicon and PS powder after 4 min of etching is presented in Figure 1, respectively. The surface of the powdered silicon is generally layered and smooth. In contrast, the surface of the PS powder was rougher, revealing highly agglomerated particulates. This indicates that etching of the silicon powder results in an increase in the surface area of the resulting PS powder. BET analysis of the PS powder showed that the surface area is ~3.10 m^2^·g^−1^. Under the conditions employed, a high surface area of the PS was not achieved.

Various mechanisms have been proposed to explain the whole process. Basically, the mechanism in which etching occurs depends on several factors, such as the method or oxidant employed for etching [17] or the proportion of the chemicals used, such as HF and HNO_3_ [18]. In this work, the etching of silicon in the HF/HNO_3_/H_2_O system may follow a chemical process with several basic reaction steps, as shown in Equations (1)–(4) [17]. Typically, etching commences with the removal of SiO_2_, which is etched away by HF to form water-soluble H_2_SiF_6_ (Equation (2)). This occurs isotropically; hence, it can result in the formation of pores. Subsequently, the oxidant, which is HNO_3_, plays an important role in ensuring the continuation of the etching process. Its function is to produce holes in the valence band of Si. This is shown in Equation (2). As a consequence, Si atoms can be removed according to Equations (3) and (4), which is based on the Gerischer mechanism [19]. The Si^+^ species reacts with HF_2_^−^, which is several times more reactive when compared to HF [19]:

(1) $$\text{Si}{\text{O}}_{2}+6\text{HF}\to {\text{H}}_{2}\text{Si}{\text{F}}_{6}+2{\text{H}}_{2}\text{O}$$

(2) $$\text{Si}+{{\text{O}}_{x}}^{+}\to {\text{Si}}^{+}+{\text{O}}_{x}$$

(3) $${\text{Si}}^{+}+3\text{H}{{\text{F}}_{2}}^{-}\to \text{HSi}{\text{F}}_{3}+2{\text{H}}^{+}+{\text{F}}^{-}+{\text{e}}^{-}$$

(4) $$\text{HSi}{\text{F}}_{3}(\text{aq})+3\text{HF}(\text{aq})\to {\text{H}}_{2}\text{Si}{\text{F}}_{6}(\text{aq})+{\text{H}}_{2}(\text{g})$$

### 2.2. Synthesis of Cu-PS

The as-synthesized PS powder was loaded with Cu particles via *in situ* reduction of Cu salt in the presence of HF. Differences in the color of the solution before and after Cu particles were loaded onto the PS powder were observed. It was found that upon addition of the PS powder to a solution of Cu salt in HF, the blue color of the salt solution changed to colorless. This was observed after separating the PS via centrifugation of the colloidal sample and is indicative that the Cu^2+^ ions are deposited and may be reduced to Cu° onto the PS powder. However, the exact nature in which the particles exist is unknown. Previous works have reported that PS can act as a reducing agent in the presence of H_2_O for metal ions, such as Pd^2+^ [20], Pt^2+^ and Au^3+^ [21], as well as Cu^2+^ [22,23]. In other words, in the presence of PS and H_2_O, Cu^2+^ ions can be reduced to Cu°. However, this was not observed or occurred only to a minimum extent in this work, as the blue color of the Cu salt solution only slightly faded without the presence of HF. This can be explained as being due to the oxidation of silicon, which easily occurs and is unable to act as a reducing agent. In this work, in the presence of HF, SiO_2_ is etched into the solution in the form of SiF_6_^2−^, as in Equation (1), leaving a fresh Si surface. Cu deposition can than occur at the expense of the surface Si. The Cu^2+^ acts as an oxidant and injects holes into the Si valance band, releasing electrons that are used for the reduction of Cu^2+^, as in Equations (2) and (5). According to previous work [24], oxidants with a more positive standard electrode potential than +0.7 V can cause this effect. Considering that the standard potential of Cu^2+^ is low, its affect as an oxidant is also low:

(5) $${\text{Cu}}^{2+}+2{\text{e}}^{-}\to \text{Cu}{}^{\circ}+0.34\;\text{V}$$

Atomic absorption spectroscopy (AAS) was conducted to investigate the extent of Cu deposition on the PS support. Results show that only 3.3 wt%, 7.8 wt% and 9.8 wt% of Cu were available on the samples prepared with 5 wt%, 10 wt% and 15 wt% of Cu, respectively. The difference in the real weight percentage when compared to the theoretical amount may be explained as being due to the poor effect of Cu^2+^ as an oxidant (hence, electron transfer from the valence band of Si to Cu^2+^ is slow) and/or due to leaching of Cu^2+^/Cu° during the preparation stage. As a note, samples in the following discussion will be referred to based on their calculated AAS values.

**Figure 2:**
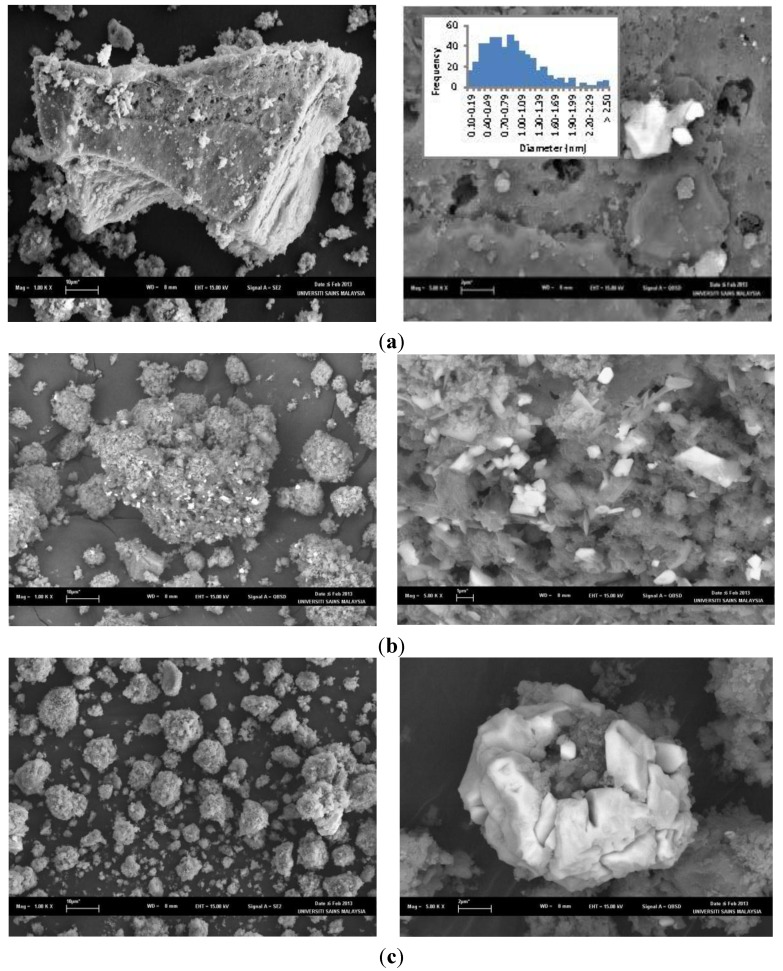
SEM images of Cu particles deposited on PS powder with a metal loading of (**a**) 3.3 wt%, (**b**) 7.8 wt% and (**c**) 9.8 wt%.

**Figure 3:**
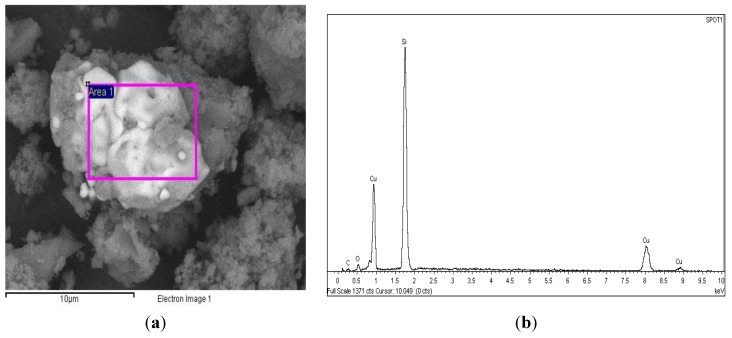
SEM-EDX analysis for a typical Cu-PS powder sample. (**a**) SEM micrograpgh; (**b**) EDX spectrum of the boxed area in (**a**).

SEM images of the Cu-PS powder samples containing various Cu content are presented in Figure 2. The Cu particles are characterized by the brighter area in the images, while the PS powder by the darker areas. Energy Dispersive X-ray (EDX) analysis of a typical image presented in Figure 3 confirms this.

As can be seen in Figure 2a, for the sample with 3.3 wt% metal loading, a number of facet-shaped particles are observed at low magnifications. At higher magnification, it also exhibits a number of small particles. These particles are a mixture of cubic-shaped and faceted particles. Therefore, two different sizes of particles exist. The average particle size is about 0.9 ± 0.5 µm. It is obvious that a bimodal size distribution exists. This is shown in the insert of Figure 2a. This occurs due to the different nucleation and growth stages of the Cu particles on the PS powder. The low metal loading on PS results in well-dispersed particles, but because of the limited metal loading, some of the particles were small, while others were larger. In contrast, when 7.8 wt% of Cu was incorporated into PS, the dispersity of the metal particles on the PS support is high and less aggregated. This is shown in Figure 2b, when the sample was viewed at higher magnifications, it exhibits a number of cube-shaped particles of almost a similar size. This may be because the increase of the metal loading from 3.3 wt% to 7.8 wt% may have allowed even the growth of the Cu nucleates and resulted in similar particle sizes. The average particles size of these particles is 4.1 ± 1.0 µm. As shown in Figure 2c, the agglomeration of Cu particles on PS occurred when the Cu loading increased to 9.8 wt%. High metal loading leads to poor dispersion and causes the affinity between the metal particles to increase [25]. This caused an increase in particle size and, finally, the aggregation of the metal particles.

BET analyses were also conducted on the Cu-PS samples to study the influence of Cu particles on the surface area of the PS support. As shown in Table 1, the surface area of PS increases upon the addition of Cu particles. The surface area of 3.3 wt% Cu-PS is 5.42 m^2^·g^−1^, and this is quadrupled to 20.52 m^2^·g^−1^ at a loading of 7.8 wt% Cu. Nevertheless, the surface area of PS experiences a sharp decrease to 3.51 m^2^·g^−1^ with a further increase in Cu loading of 9.8 wt%. The initial increment in surface area may be due to the phenomenon of metal-assisted chemical etching, while the latter decrement is caused by the particle agglomeration. Cu particles have been used as catalyst to assist in chemical etching of Si [26]. In this study, Cu particles are formed via the redox reaction (see Equations (2) and (5)) in the presence of HF. The Si formed near the Cu particles holes. Hence, this leads to the surface area of Cu-PS with 3.3 wt% and 7.8 wt% Cu being higher than neat PS. Nonetheless, when Cu particles are highly agglomerated on the PS at higher loading (*viz*. 9.8 wt% Cu), this can cause a blockage of the pores for the etching process. Therefore, the surface area for 9.8 wt% Cu-PS drops drastically and approaches neat PS.

**Table 1:** Surface area, particle size of Cu°, H_2_ consumption and production for various samples.

Notation	Cu° Content * (wt%)	BET Surface Area (m^2^·g^−1^)	Cu° Particle Size	H_2_ Consumed (mol·g_metal_^−1^)	H_2_ Produced (mol·g_metal_^−1^)
PS	0	3.10	N/A	Nil	Nil
3.3 wt% Cu-PS	3.3	5.42	0.9 ± 0.5 μm	5.70 × 10^−3^	3.27 × 10^−3^
7.8 wt% Cu-PS	7.8	20.52	4.1 ± 1.0 μm	4.41 × 10^−3^	3.33 × 10^−4^
9.8 wt% Cu-PS	9.8	3.51	Highly agglomerated	2.88 × 10^−3^	9.59 × 10^−4^

* Determined via AAS.

### 2.3. H_2_-TPR Analysis of Cu-PS

**Figure 4:**
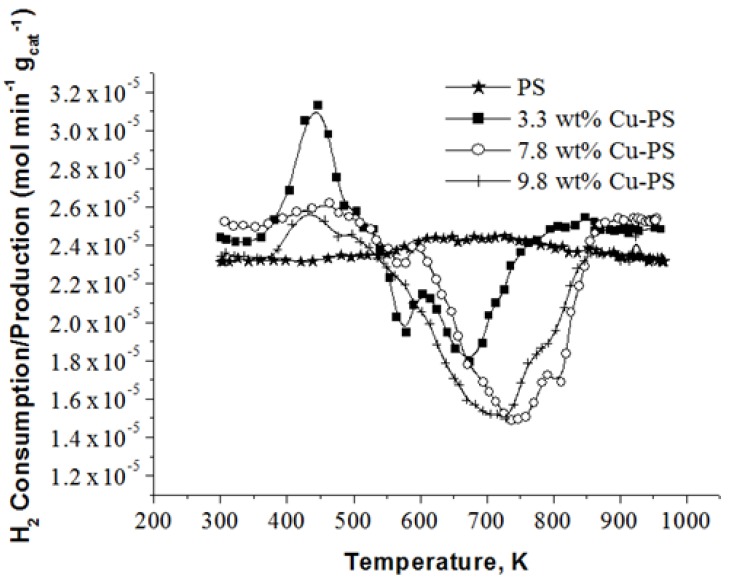
H_2_-temperature program reduction (TPR) profiles of the PS and various wt% of Cu-PS powder samples.

The H_2_-TPR profiles of PS and the various Cu-PS powder samples are displayed in Figure 4. It can be seen that the PS powder exhibits no hydrogen consumption. This is because there is no metal phase on the support. For Cu-PS catalysts, H_2_ consumption peaks are available. This is due to the availability of copper oxide, which is expected due to the fact that the synthesized Cu particles on the PS powder may be oxidized upon exposure to air. The position of the H_2_ consumption peaks available can reflect: (i) the type of CuO reduction reaction, which may occur (a one-step or two-step process) [27,28,29]; or (ii) the various Cu particle sizes available [29]. Considering that the H_2_ consumption occurred at a temperature above 570 K in all of the catalysts containing Cu, it is possible to infer that the reduction of CuO occurred via a two-step process [28,30]. Hence, the difference in the positions of the H_2_ consumption peaks is attributed to the variation in particle size. The H_2_-TPR profiles for 3.3 wt% Cu-PS exhibit consumption peaks at 575 K and 673 K. The peak centered at 575 K may be due to the reduction of highly-dispersed copper oxide particles, which are smaller in size, whereas the peak centered at 673 K may be due to the larger or aggregated copper oxide. The lower metal loading on the support may have led to the higher dispersion. This is confirmed by SEM images. Chang and coworkers [25] reported that supported samples with low metal loading are easier to reduce. This implies that lower metal loading exerts a weak metal-support interaction (MSI). However, this largely depends on the weight percentage of the metal on the support, as well as the size of the particles, as demonstrated by Aguila and coworkers [29].

**Figure 5:**
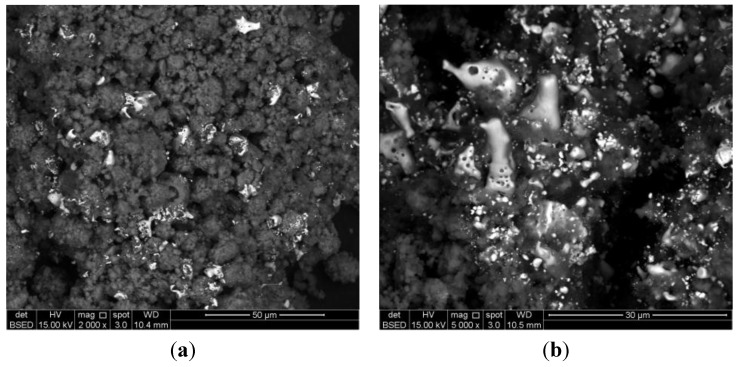
SEM images of the 7.8 wt% Cu-PS sample after H_2_-TPR analysis at magnifications of (**a**) 2000×; (**b**) 5000×.

The H_2_-TPR profile for 7.8 wt% Cu-PS exhibits a H_2_ consumption peak at 744 K with a shoulder at approximately 820 K. This is due to the presence of larger metal particle sizes when compared to 3.3 wt% Cu-PS. The shoulder at 820 K indicates the existence of even bigger Cu particles. In comparison, the H_2_-TPR profile for 9.8 wt% only exhibits one peak at 711 K and a slight shoulder at 783 K. The similar characteristics and position of this peak with the H_2_ consumption peak that arises in the 7.8 wt% Cu-PS sample indicates similarities in the particle size or availability of aggregates. It should be noted that this finding contradicts the SEM results, where no serious aggregations were observed in the 7.8 wt% Cu-PS sample. Nevertheless, particle aggregation may have occurred upon heat treatment of the 7.8 wt% Cu-PS sample during the H_2_-TPR analysis. This is shown in Figure 5, whereby large aggregates similar to that observed in the sample containing 9.8 wt% of Cu are seen. With the supply of external energy from heat, the smaller particles may have easily fused together, grew into a larger size and aggregated, giving rise to the H_2_-TPR profiles observed. The occurrence of peaks at high temperature in both the 7.8 wt% and 9.8 wt% samples when compared to the 3.3 wt% Cu-PS samples also indicates the fact that particle sizes are larger and the MSI is weak. As such, the particles are harder to reduce.

**Figure 6:**
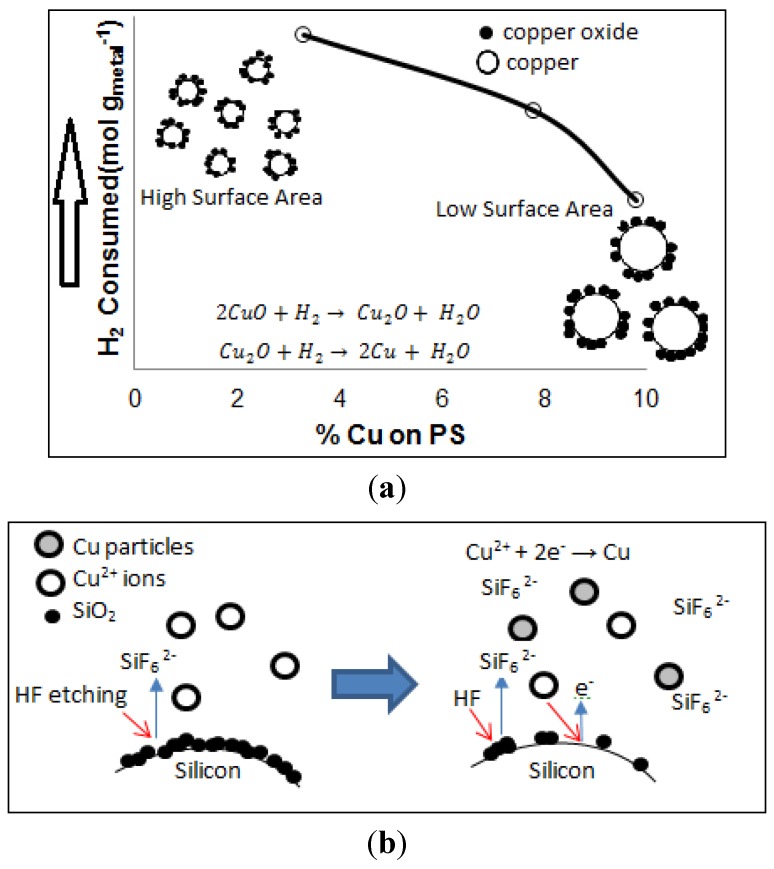
Schematic diagrams for (**a**) H_2_ consumption and (**b**) H_2_ production reactions.

The amount of H_2_ consumed during the H_2_-TPR analysis was also calculated. It is observed that H_2_ consumption decreases with increasing Cu content in PS. The H_2_ consumption is 5.70 × 10^−3^ mol·g_metal_^−1^ for the 3.3 wt% Cu-PS, and this is decreased to 4.41 × 10^−3^ and 2.88 × 10^−3^ mol·g_metal_^−1^ for a Cu loading of 7.8 wt% and 9.8 wt%, respectively. Thus, the Cu particles are less oxidized in the sample with higher Cu content. It is inferred that the oxidation of Cu mainly occurs on the surface of the particles. Therefore, larger particles with a lower surface area give rise to lower H_2_ consumption. This is depicted in Figure 6a.

An interesting observation in all samples is that H_2_ production is observed in all of the Cu-PS samples. This occurs at approximately 444 K. The production of H_2_ is 3.27 × 10^−3^, 3.33 × 10^−4^ and 9.59 × 10^−4^ mol·g_metal_^−1^ for PS incorporated with 3.3 wt%, 7.8 wt% and 9.8 wt% Cu, respectively. A possible explanation for the production of H_2_ is that H^+^ or H_2_ formed during the reduction of Cu ions may have adsorbed onto the Cu particles upon their formation, as in Figure 6b. This is then desorbed during the TPR analysis. The different amounts of H_2_ produced by the Cu-PS samples may be related to the size of the particles, as well as the various sites available on the Cu surface. It is observed that the amount of H_2_ production decreases drastically when the amount of Cu incorporated onto PS is increased from 3.3 wt% to 7.8 wt%. This can be explained as being due to the larger size of the Cu particles available when 7.8 wt% of Cu is incorporated onto the PS. The larger Cu particle size has a lower surface area, which is exposed for H^+^ or H_2_ to adsorb initially. In contrast, when the Cu content is further increased to 9.8 wt%, H_2_ production also increases with respect to the sample containing 7.8 wt% of Cu. In this case, the higher H_2_ production is attributed to the availability of different active sites on the Cu particles. This is confirmed by the existence of an extra peak positioned at approximately 500 K in the 9.8 wt% Cu-PS, which is not available in samples containing 3.3 wt% and 7.8 wt% of Cu.

### 2.4. Catalytic Test

**Figure 7:**
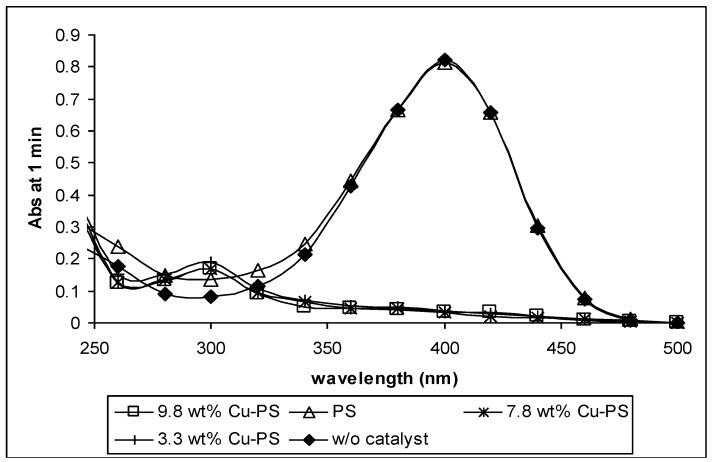
UV spectra of solution containing p-nitrophenol and KBH_4_ with (PS or Cu-PS) and without catalysts at 1 min of reaction.

The effectiveness of Cu-PS in the catalytic reduction of p-nitrophenol was monitored by a ultraviolet-visible spectrophotometer, and the absorption spectra are presented in Figure 7. The mixture of p-nitrophenol and KBH_4_ shows a brilliant yellow color that gives a broad UV peak at 400 nm due to the charge transfer absorption of the 4-nitrophenolate ions [31]. The reduction of p-nitrophenol occurs instantaneously in the presence of Cu-PS powder, regardless of the Cu loading, whereby the yellowish solution turns colorless within a minute. The disappearance of the absorption peak at 400 nm was accompanied by the formation of a new absorption peak at ~300 nm in the spectrum. These observations suggest that p-nitrophenol was successfully reduced to p-aminophenol [32]. In the case of the PS powder sample, a similar UV profile as the mixture of p-nitrophenol and KBH_4_ was observed after a minute of reaction (Figure 7). Nevertheless, the discoloration of the solution occurred gradually and became colorless after about an hour. UV spectra of the gradual decrement in intensity of the peak at 400 nm were also recorded at several time interval during the hour. However, this is not shown here. Thus, it is envisaged that the PS powder catalyzes the reduction of p-nitrophenol, but in a much lower efficiency, as compared to Cu-PS powder samples. Other works have also shown catalytic reductions of p-nitrophenol, which can occur within 15 min; however, mostly in these works, noble metals, such as 1 wt% Au/Al_2_O_3_ [32], Ag-deposited silica-coated Fe_3_O_4_ magnetic particles [31], hierarchical silver microstructures [33] and Pd stabilized poly(vinylpyrrolidone) (PVP) [34], were employed.

Generally, the catalytic activity of metal catalyst is dependent on its size, shape and composition. In this study, the catalytic reduction of p-nitrophenol to p-aminophenol by the Cu-PS powders may not only be affected by the PS itself and the morphology of the Cu particles on the PS, but also due to the availability of CuO. Previous works have shown that CuO is also effective at the catalytic reduction of aromatic nitro compounds [35]. Although we have shown that PS can result in the catalytic reduction of p-nitrophenol to p-aminophenol, in this work, its contribution is small. Hence, here, the conversion of p-nitrophenol is largely attributed to the availability of Cu and CuO. Further works are being conducted to investigate how the composition of Cu and CuO influences the catalytic reactivity. In this work, the difference in the catalytic activity of the Cu-PS powders with different wt% of Cu was unobservable. We believe that this may be attributed to the following reasons: first, the difference (if any) in the reaction time is hard to detect due to fast reduction (*viz*. < 1 min), regardless of the metal loading; second, the agglomeration of Cu particles may have broken down by agitation during reaction.

## 3. Experimental Section

### 3.1. Materials

Copper sulfate, CuSO_4_·5H_2_O, was purchased from R&M Chemicals (Essex, UK). Silicon powder (99.99% purity with an average grain size of 325 mesh) was obtained from Aldrich (Steinheim, Germany), while hydrofluoric acid, HF (49 wt%), and nitric acid, HNO_3_ (69 wt%), were obtained from QReC (Selangor, Malaysia). Purified argon (99.995%) and purified hydrogen (99.995%) were supplied by MOX-Linde Gases Sdn. Bhd. (Penang, Malaysia), while diluted hydrogen (99.999%) was from Air Liquide (Selangor, Malaysia). All chemicals and gasses were used as received.

### 3.2. Methods

#### 3.2.1. Synthesis of Porous Silicon (PS) Powder

The PS powder was prepared by the chemical etching method in a mixture of HF/HNO_3_/H_2_O (1/3/5 v/v). In a typical preparation; 1.5 g of silicon powder was weighed and placed in a plastic beaker. Then, 5 mL of HF, 15 mL of HNO_3_ and 25 mL of distilled water, respectively, were added. The mixture was stirred vigorously for 4 min at room temperature. After stirring, the mixture was centrifuged for 5 min at 3500 rpm. The supernatant was discarded, and the etched powder silicon was rinsed several times with distilled water. Finally, the PS powder was dried at 343 K for 24 h and kept in a desiccator until further analysis.

#### 3.2.2. Synthesis of Copper Deposited PS (Cu-PS) Powder

The Cu salt is reduced *in situ* in the presence of PS. A certain amount of aqueous 0.1 M CuSO_4_·5H_2_O was pipetted into a round-bottom flask. This was followed by the addition of 1 mL HF. Then, as much as 1.0 g of the as-prepared PS powder was added to the flask, while stirring for ~10 min at room temperature. Decoloration of the initial blue-green solution is instantaneous and signifies the reduction of Cu ions. The copper metal-incorporated PS (Cu-PS) powder was then centrifuged and washed repeatedly with distilled water. The Cu-PS powder was dried in a vacuum oven at 343 K for 24 h. Other Cu-PS powders of various amounts of metal loadings were prepared accordingly.

#### 3.2.3. Catalytic Reduction of P-Nitrophenol

In a sample vial, 25.0 mg of KBH_4_ was weighed carefully and added with 20 mL of distilled water for dilution. As much as 5 mg of catalyst were then added to the KBH_4_ solution. The reaction starts once 20 μL of 4.66 × 10^−2^ M p-nitrophenol were introduced. The reduction of p-nitrophenol was monitored with a Hitachi UV-Vis spectrophotometer (Tokyo, Japan).

### 3.3. Characterizations

The morphology of the samples were examined by SEM using a FESEM LEO SUPRA 50 VP (Carl-Ziess-SMT, Oberkochen, Germany). A small portion of the powder sample was placed on carbon tape on a SEM plate and coated with platinum. Several images were taken from random locations to ensure that the images recorded were representative as a whole. The BET surface area of samples was measured from N_2_ adsorption isotherms with a Surface Area and Porosity Analyzer (ASAP2020, Micromeritics, Norcross, GA, USA). Prior to this measurement, the samples were dried overnight in an oven at 403 K and then quickly placed in a sample tube under N_2_ atmosphere. After that, the tube was heated to 500 K and vacuum evacuated. Finally, N_2_ gas is redirected to the sample. The H_2_-TPR analysis was carried out to investigate the reducibility of the Cu-PS samples. As much as 200 mg of the sample were weighed and placed in a U-tube reactor. Diluted hydrogen gas with a flow rate of 100 mL·min^−1^ was then passed through the reactor while heating from room temperature to 973 K at a rate of 5 K·min^−1^. The resulting effluent gas was analyzed every 2 min using a G2890A microchromatograph (Inficon, Cambridge, MA, USA) operating at 303 K. AAS analysis was carried out with a Perkin-Elmer Analyst 200 (Perkin Elmer, Waltham, MA, USA). The samples were prepared by digesting 50 mg of the Cu-PS in aqua regia. The digested sample was diluted to 50 mL with distilled water. As much as 1 mL of the sample was further diluted with distilled water in a 25-mL volumetric flask. This sample was filtered before transferring into a plastic bottle for measurement. The AAS measurement procedure was repeated in triplicate. The AAS results are expressed in terms of wt% of Cu, as tabulated in Table 1.

## 4. Conclusions

The PS powder was synthesized in an etching solution of HF:HNO_3_:H_2_O (1:3:5 v/v). The SEM images show that the surface of powdered silicon upon etching is porous when compared to the surface of powdered silicon before etching. The effects of the deposition of different percentages of Cu on the PS have also been reviewed by using H_2_-TPR, SEM and AAS. SEM images show that there are different sizes of Cu particles on the PS when the PS was impregnated with the different weight percentages of metal salt. Based on this analysis, it is shown that low percentages of Cu lead to the formation of mostly small-sized cubic particles due to the limited amount of metal loading. When the percentage of metal loading is increased from 3.3 wt% to 9.8 wt%, the metal particles tend to grow, as well as become aggregated. H_2_-TPR studies also support these findings. All of the Cu-PS powders show excellent catalytic activity for the reduction of p-nitrophenol with a reduction time of <1 min.
